# A high serum-free fatty acid level is associated with cancer

**DOI:** 10.1007/s00432-019-03095-8

**Published:** 2019-11-26

**Authors:** Lili Zhang, Lei Han, Juan He, Jing Lv, Rongfang Pan, Teng Lv

**Affiliations:** 1 Department of Nutrition, The Affiliated Hospital of Qingdao University, Qingdao, 266000 China; 2 Department of Gynaecology, The Affiliated Hospital of Qingdao University, 1677, Wutaishan Road, Xihaian District, Qingdao, 266000 China

**Keywords:** Free fatty acids, Cancer, Cancer biomarker, Early diagnosis, Malignant neoplasm

## Abstract

**Purpose:**

The objectives of this work were to investigate whether the serum-free fatty acid (FFA) level is meaningful in cancer patients and its role in cancer diagnosis.

**Methods:**

A total of 2206 patients were divided into a cancer group (*n* = 1019) and a noncancer group (*n* = 1187). Age, sex, body mass index (BMI), and serum FFA and serum albumin levels were collected. Cancer patients were divided into subgroups according to the location of the cancer. We then compared serum FFA levels among the tumor subgroups. A receiver operating characteristic (ROC) curve analysis was performed to further evaluate the diagnostic ability of the FFA level. SPSS 22.0 software was used to analyze the results.

**Results:**

The FFA level was higher in the cancer group than in the noncancer group. According to the multivariate analysis, there was also an increased risk of cancer associated with a high FFA level after adjusting for old age, female sex, and a low BMI. In the subgroup analysis, the FFA level in patients with lung cancer, gastric cancer, thyroid cancer, rectal cancer, colon cancer, and ovarian cancer was significantly higher than that in noncancer patients. The area under the effect–time curve (AUC) of FFAs in the whole cancer group was 0.58, while the thyroid cancer, rectal cancer, and ovarian cancer subgroups had AUCs > 0.6.

**Conclusion:**

Our study provides clinical evidence to support that fatty acid metabolism is associated with cancers and demonstrates that a high FFA level in the serum may be an indicator of cancer.

## Introduction

It is estimated that there were 18.1 million new cases and 9.6 million cancer deaths worldwide in 2018 according to global cancer statistics (Bray et al. 2018). In 2018, the China Cancer Center released the National Cancer Report, which showed that in 2014, the estimated number of new cases of cancer in China was 38.04 million (21.14 million males and 16.99 million females) (Chen et al. 2018). Cancer has become one of the most crucial problems threatening human health. Cancer stage is directly related to mortality (Edwards et al. 2014; Howlader et al. 2014; Dianatinasab et al. 2018). Presymptomatic screening is related to early stage diagnosis and improved outcomes (Plumb et al. 2016; Inari et al. 2017). By increasing the cancer screening rates in those who are most likely to develop cancer, we can provide them with a definite opportunity to reduce mortality (Stich and Berlan 2004). However, most cancer patients are diagnosed at an advanced stage due to a lack of symptoms and limitations in currently available tumor markers. Free fatty acids (FFAs) are intermediate products of lipid mobilization that result principally from lipolysis and the breakdown of triglycerides (TGs) (Stich and Berlan 2004). Previous studies have reported that FFA levels are associated with unfavorable functional outcomes in stroke and cardiovascular disease and may play a role in the process of disease progression (Choi et al. 2014; Sun et al. 2015; Xiong et al. 2015; Duan et al. 2017; Han et al. 2018). In clinical work, we found that in some cancer patients, the FFA level was elevated even when TGs and total cholesterol (TCHO) were normal, and a high FFA level may be the only abnormality in biochemical tests. To identify whether there are differences in the FFA level between cancer and noncancer patients and the role of FFAs in the diagnosis of cancer, we designed the study described herein. If the FFA level is an indicator of cancer, patients with a high FFA level may benefit from increasing the cancer screening rates and follow-up tests.

## Materials and methods

### Patients

From January 2019 to June 2019, patients aged 18–65 years with normal blood lipids (serum TGs 0.3–1.92 mmol/L, TCHO 2.32–5.62 mmol/L, and low-density lipoprotein cholesterol 1.9–3.12 mmol/L), normal liver, and kidney function (alanine aminotransferase 7–40 U/L, aspartate aminotransferase 13–35 U/L, urea nitrogen 3.1–8.8 mmol/L, and creatinine 31–132 µmol/L) were extracted from the electronic medical record system of the Affiliated Hospital of Qingdao University. Patients with diabetes mellitus, hyperlipidemia, abnormal thyroid function, stroke, or cardiovascular disease and those who were pregnant were excluded. We collected body weight and height data, and the BMI (body mass index) was calculated. Depending on the diagnosis, the patients were divided into a cancer group and a noncancer group. The cancer group had definite pathological or imaging evidence, while the noncancer group did not. Tumors that could not be identified as benign or malignant were not included in any group. The general data of the two groups are shown in Table 1.

**Table 1:** Patient characteristics

Characteristic	No. of cancer patients (*n* = 1019)	No. of noncancer patients (*n* = 1187)	*t* or *χ*^2^ (*P* value)
Sex			
Male [*n* (%)]	438 (43%)	730 (62%)	< 0.001
Female [*n* (%)]	581 (57%)	457 (38%)	< 0.001
Average age (years)	52.62 ± 9.11	47.85 ± 13.20	< 0.001
BMI (kg/m^2^)	24.29 ± 4.89	24.94 ± 3.62	< 0.001
Laboratory findings			
FFA (mmol/L)	0.45 (0.33–0.59)	0.41 (0.29–0.56)	< 0.001
Albumin (mmol/L)	42.41 (39.78–44.94)	40.56 (36.04–44.60)	< 0.001

Average age and BMI are presented as the mean value ± standard deviation (SD), and serum FFA and albumin levels are presented as the median value (25th percentile, 75th percentile)

### Laboratory testing

The GPO–PAP method was adopted to determine the TG level. The CHOD–POD method was adopted to determine the TCHO level. The enzyme endpoint method was applied to measure the FFA level. The FFAs that we measured were nonesterified fatty acids. Serum albumin was detected by spectrophotometry with bromocresol green. We collected only the first FFA and albumin measurements after admission. The biochemical analyzer used was Beckman Coulter AU5800 (USA).

### Statistical analysis

We used the one-sample Kolmogorov–Smirnov test to examine the normality of continuous variables. The concentrations of serum FFA and albumin were naturally logarithmically transformed to improve normalization. The results are presented as percentages for categorical variables and as the mean value ± SD or as the median value (25th percentile, 75th percentile) for continuous variables. Differences in continuous variables were compared using Student’s *t* test (normal distribution) or the Mann–Whitney *U* test (skewed distribution), and the distributions of categorical variables were compared using the Chi-square test. Correlations among continuous variables were assessed by the Spearman rank correlation coefficient.

The odds ratios (ORs) and 95% confidence intervals (CIs) of the serum FFA concentration between cancer and noncancer patients were evaluated by univariate or multivariate logistic regression analyses after adjusting for age, sex, BMI, and albumin concentration. A receiver operating characteristic (ROC) curve analysis was performed to further evaluate the diagnostic ability of the FFA level. All statistical analyses were performed with SPSS software (version 22.0, Chicago, IL, USA). Statistical significance was accepted at the *P* ≤ 0.05 level. AUC values of ≤ 0.5, 0.5 to < 0.7, 0.7 to < 0.8, and 0.8 to < 0.9 and ≥ 0.9 indicate no, poor, acceptable, excellent and outstanding discrimination, respectively.

## Results

In our study, serum FFAs were collected from 2206 patients. Overall, 1168 patients (53%) were male and had a median age of 50 years. Patients were divided into two groups: a cancer group and a noncancer group. The main baseline characteristics of the patients are summarized in Table 1. The cancer patients were older than the noncancer patients(52.62 vs 47.85 years, *P* < 0.001), and the proportion of males in cancer patients was lower than that in noncancer patients (43% vs 62% male, *P* < 0.001). The BMI in the cancer group was 24.29 kg/m^2^, which was lower than that in the noncancer group (24.94 kg/m^2^, *P* < 0.001). Compared with the noncancer group, the cancer group had a higher FFA level (0.45 vs 0.41 mmol/L, *P* < 0.001) and a higher albumin level (42.41 vs 40.56 mmol/L, *P* < 0.001).

As shown in Table 2, the univariate analysis revealed that old age, female sex, a low BMI, and high FFA and albumin levels were associated with an increased risk of cancer. After adjusting for age, sex, BMI, and albumin, each unit of the ln-transformed FFA values generated a 35.8% risk of cancer [OR (95% CI): 1.358 (1.126, 1.638), *P* < 0.001].

**Table 2:** Univariate and multivariate logistic regression analyses between groups

Parameter	Univariate analysis			Multivariate analysis		
	OR	95% CI	*P* value	OR	95% CI	*P* value
Age	1.037	1.029–1.045	< 0.001	1.065	1.055–1.075	< 0.001
Female	2.199	1.787–2.531	< 0.001	2.237	1.859–2.692	< 0.001
BMI	0.954	0.931–0.978	< 0.001	0.922	0.896–0.948	< 0.001
FFA	1.56	1.315–1.850	< 0.001	1.358	1.126–1.638	< 0.001
Albumin	1.076	1.059–1.094	< 0.001	1.131	1.109–1.154	< 0.001

FFA and albumin levels were ln-transformed in the models

We divided the cancer patients into subgroups according to the location of the cancer. We then divided the patients into subgroups (*n* ≥ 20 per subgroup). Table 3 shows that patients with thyroid cancer have higher FFA levels, while patients with breast cancer have lower FFA levels. Table 4 shows that there was a significant positive correlation between FFA and BMI in noncancer and breast cancer patients. For each additional unit of BMI, the FFA level increases by 1% in noncancer patients and by 3.4% in breast cancer patients.

**Table 3:** Comparison of serum FFA levels among tumor subgroups

Group	*n*	FFA (mmol/L)	Other cancers	*P*	BMI	Other cancers	*P*
Lung cancer	288	0.44 (0.32–0.59)	0.46 (0.34–0.60)	0.258	24.81 ± 7.56	24.08 ± 3.29	0.035
Breast cancer	197	0.42 (0.27–0.55)	0.48 (0.36–0.63)	< 0.001	24.49 ± 3.20	24.30 ± 3.12	0.497
Gastric cancer	146	0.46 (0.35–0.60)	0.45 (0.33–0.59)	0.493	23.26 ± 3.21	24.46 ± 5.10	0.006
Thyroid cancer	106	0.50 (0.38–0.60)	0.45 (0.32–0.59)	0.039	24.58 ± 3.34	24.25 ± 5.05	0.521
Rectal cancer	72	0.50 (0.37–0.64)	0.45 (0.33–0.59)	0.117	23.72 ± 2.68	24.33 ± 5.02	0.315
Colon cancer	66	0.48 (0.35–0.61)	0.45 (0.33–0.59)	0.246	24.59 ± 3.43	24.27 ± 4.98	0.609
Esophageal cancer	46	0.41 (0.30–0.58)	0.45 (0.33–0.59)	0.299	21.90 ± 3.05	24.40 ± 4.94	< 0.001
Cervical cancer	40	0.47 (0.38–0.60)	0.46 (0.33–0.60)	0.413	24.28 ± 2.55	24.37 ± 3.19	0.861
Ovarian cancer	20	0.50 (0.41–0.60)	0.45 (0.33–0.60)	0.144	25.30 ± 3.24	24.33 ± 3.14	0.174

The BMI and FFA levels were compared among females with breast cancer, cervical cancer, ovarian cancer and other cancers

**Table 4:** Relationship between FFA and BMI in the univariate regression model

Group	*n*	*β* (95% CI)	*P*
Noncancer	1187	0.010 (0.002, 0.018)	0.020
Lung cancer	288	0.004 (− 0.004, 00.012)	0.287
Breast cancer	197	0.034 (0.011, 0.057)	0.005
Gastric cancer	146	− 0.007 (− 0.030, 0.016)	0.550
Thyroid cancer	106	− 0.011 (− 0.031, 0.010)	0.313
Rectal cancer	72	− 0.002 (− 0.039, 0.035)	0.896
Colon cancer	66	0.026 (− 0.010, 0.062)	0.149
Esophageal cancer	46	− 0.000 (− 0.046, 0.045)	0.990
Cervical cancer	40	0.031 (− 0.017, 0.078)	0.195
Ovarian cancer	20	− 0.001 (− 0.042, 0.040)	0.959

FFA levels were ln-transformed in the univariate regression model

The Mann–Whitney *U* test indicated that the FFA levels in patients with lung cancer, gastric cancer, thyroid cancer, rectal cancer, colon cancer, and ovarian cancer were significantly higher than those in noncancer patients (Table 5) (*P < *0.05).

**Table 5:** Comparison of FFA levels between each cancer subgroup and noncancer patients

	*n*	Q1	Median	Q3	*P*
Noncancer	1187	0.29	0.41	0.56	–
Lung cancer	288	0.32	0.44	0.59	0.017
Breast cancer	197	0.27	0.42	0.55	0.351
Gastric cancer	146	0.35	0.46	0.60	0.003
Thyroid cancer	106	0.38	0.50	0.60	< 0.001
Rectal cancer	72	0.37	0.50	0.64	0.001
Colon cancer	66	0.35	0.48	0.61	0.007
Esophageal cancer	46	0.30	0.41	0.58	0.653
Cervical cancer	40	0.38	0.47	0.60	0.084
Ovarian cancer	20	0.41	0.50	0.61	0.028

*Q 1* first quartile, *Q3* third quartile

The FFA levels were compared among female breast cancer, cervical cancer, ovarian cancer and noncancer patients

Table 6 shows that the ROC curve yielded an AUC of 0.561 (95% CI 0.52–0.56) in the whole cancer group, demonstrating that the FFA level had poor discriminative ability to distinguish cancer patients from noncancer patients. In the subgroup analysis, all of the cancer subgroups yielded AUCs of > 0.5, indicating that the FFA level had poor diagnostic value in lung cancer, breast cancer, gastric cancer, thyroid cancer, rectal cancer, colon cancer, esophageal cancer, cervical cancer, and ovarian cancer. Among them, thyroid cancer, rectal cancer, and ovarian cancer had AUCs of > 0.6.

**Table 6:** AUC values, cut-off values, sensitivity, and specificity of FFA levels between cancer subgroups and noncancer patients

Subgroup	AUC (95% CI)	Sensitivity (%)	Specificity (%)	Cut-off
Whole cancer	0.561	0.713	0.390	0.355
Lung cancer	0.545	0.590	0.500	0.405
Breast cancer	0.523	0.843	0.232	0.605
Gastric cancer	0.576	0.753	0.370	0.345
Thyroid cancer	0.620	0.670	0.563	0.435
Rectal cancer	0.614	0.556	0.648	0.485
Colon cancer	0.599	0.439	0.731	0.545
Esophageal cancer	0.520	0.130	0.939	0.765
Cervical cancer	0.582	0.800	0.381	0.365
Ovarian cancer	0.645	0.95	0.435	0.385

The AUCs were compared among female patients with breast cancer, cervical cancer, and ovarian cancer

## Discussion

In the present study, we investigated whether the FFA level was meaningful between cancer and noncancer patients and found that the FFA level was higher in cancer patients than in noncancer patients. These findings indicate that the FFA level is pathogenically involved in cancer, which was clearly demonstrated in several studies in related fields. Recent advances in proteomics and metabolomics have deepened our understanding of the role of fatty acid metabolism in determining the fate of cancer cells (Li et al. 2017; Wang et al. 2018; Madak-Erdogan et al. 2019). The TG/FFA cycle participate various metabolic, physiological, and signaling pathways in cells. Fatty acid metabolism not only supports energy production but also plays an important role in the biosynthesis pathway, which is crucial to neogenesis (Grierson et al. 1990). Our study provides strong clinical evidence that FFAs play a role in the process of cancer progression.

As a source of metabolic energy, a substrate for cell membrane structures, and a precursor to many intracellular signaling molecules (Grierson et al. 1990), FFAs may be affected in a number of pathological conditions, such as insulin resistance, type 2 diabetes, obesity, severe liver dysfunction, hyperthyroidism, and so on(Rui 2014; Tseng et al. 2015). It is also affected by dietary and insulin fluctuations (Xin et al. 2019). Elevated FFA levels can lead to insulin resistance and other metabolic disorders (Arner 2002; Boden 2003). According to the multivariate analysis, there is an increased risk of cancer associated with a high FFA level after adjusting for other confounders. The results suggested that an elevated FFA level is an independent risk factor for cancer, especially lung cancer, gastric cancer, thyroid cancer, rectal cancer, colon cancer, and ovarian cancer. The results also showed that there was a significant positive correlation between the FFA level and BMI in noncancer patients and breast cancer patients, while in the other cancer group, there was no significant correlation. The ROC value of the whole cancer population as a diagnostic criterion was 0.56. As a prognostic indicator, the FFA level was weak. However, thyroid cancer, rectal cancer, and ovarian cancer subgroups had AUCs of > 0.6, which was slightly higher than the other subgroups. This finding implies that an elevated FFA level may be a potential biomarker for cancers.

Some studies (Nayan et al. 2017; Yokomichi et al. 2017; Li et al. 2019a, b) have found that statin use in cancer improves survival outcomes and increases overall survival. Our results can also explain why lipid-lowering therapy can improve clinical outcomes since cancer patients have elevated FFA levels even when serum TGs are normal.

Our results are consistent with those of Zhang et al. (2014a, b, 2016), who performed chip-based direct-infusion nanoESI Fourier transform ion cyclotron resonance mass spectrometry (CBDInanoESI-FTICR MS) to simultaneously quantitatively and qualitatively analyze multiple targeted serum unsaturated FFAs. The authors found that unsaturated FFAs could be a potential biomarker panel for the early detection of some cancers.

Our research has certain limitations. First, we did not collect enough FFA data from healthy people. We collected data from patients who underwent the FFA test in the medical record system, and there may be certain deviations. Second, to avoid possible interference factors, we excluded patients with diabetes, hepatic insufficiency, renal insufficiency, thyroid diseases, coronary atherosclerotic heart disease, stroke, and pregnancy which may have affected the results. Since FFA levels might increase postoperatively, we collected only the first FFA measurement after admission, since it was obtained prior to surgery and had the fewest influencing factors. However, most of the tumor patients underwent inpatient surgery and were thus generally in a good condition to tolerate surgery, while the nontumor patients were inpatient general internal medicine patients who required hospitalization and were thus often in a poor condition. This may be one reason why surgical patients have higher levels of albumin than internal medicine patients. Our data on tumor distribution do not represent the incidence of tumors in our region. On the one hand, we selected cases with specified conditions. On the other hand, patients with different diseases have a preferential choice of hospital.

## Conclusions

In summary, high FFA levels in the serum may be an indicator of cancer, especially when they are raised because of an unknown reason.

## Data Availability

All data generated or analyzed during this study are included in this published article and its Online Resources.
